# Primary Stability in Hip Revision Arthroplasty: Comparison of the Stability of Cementless Fixed Augments on a Modular Acetabular Cage System with and without Cranial Straps

**DOI:** 10.3390/jcm10174002

**Published:** 2021-09-04

**Authors:** Max Jaenisch, Hendrik Kohlhof, Dieter Christian Wirtz, Frank Alexander Schildberg, Nicholas A. Beckmann, Jan Philippe Kretzer, Mareike Schonhoff, Sebastian Jäger

**Affiliations:** 1 Department for Orthopaedics and Trauma Surgery, University Hospital Bonn, 53127 Bonn, Germany; Hendrik.Kohlhof@ukbonn.de (H.K.); Dieter.Wirtz@ukbonn.de (D.C.W.); Frank.Schildberg@ukbonn.de (F.A.S.); 2 Laboratory of Biomechanics and Implant Research, Clinic for Orthopaedics and Trauam Surgery, Heidelberg University Hospital, 69118 Heidelberg, Germany; Nicholas.Beckmann@med.uni-heidelberg.de (N.A.B.); Philippe.Kretzer@med.uni-heidelberg.de (J.P.K.); Mareike.Schonhoff@med.uni-heidelberg.de (M.S.); Sebastian.Jaeger@med.uni.heidelberg.de (S.J.)

**Keywords:** hip, revision, arthroplasty, modular, cementless, augment, cranial strap

## Abstract

The goal of this study is to evaluate the primary stability of a cementless augment-and-modular-cage system with and without the addition of cranial straps in a standardized in vitro setting. As the surrogate parameter for the evaluation of primary stability, the measurement of relative motion between the implant components themselves and the bone will be used. Acetabular revision components with a trabecular titanium augment in combination with a large fourth-generation composite left hemipelvis were assembled. These constructs were divided into two groups with (S) and without cranial straps (nS). A total of 1000 cycles was applied at each of three load levels. Relative movements (RM) between the components were measured. Load levels display a significant effect on the amount of RM at all interfaces except between shell/augment. The group assignment appears to have an effect on RM due to significantly differing means at all interfaces. Between bone/shell RM increased as load increased. NS displayed significantly more RM than S. Between shell/augment RM remained constant as load increased. Between shell/cup S showed more RM than nS while both groups’ RM increased with load. We conclude a significant increase of primary stability between the shell and the bone through the addition of cranial straps. Relative motion between components (shell/cup) increases through the addition of cranial straps. A clinical impact of this finding is uncertain and requires further investigation. Finally, the cementless fixation of the augment against the rim-portion of the shell appears stable and compares favorably to prior investigation of different fixation techniques.

## 1. Introduction

The number of primary hip arthroplasties is rising all over the developed world [1]. Due to bone loss caused by aseptic loosening and periprosthetic infection, surgeons often encounter severe acetabular defects in cases of revision surgery. With an increased number of primary hip arthroplasties, a consecutive rise in revision arthroplasties is to be expected [2]. In order to achieve long-term stability, a primary stable implantation with proper force transmission to the remaining acetabular bone stock is essential. Therefore, non-contained acetabular defects should be transitioned into contained defects [3].

While structural bulk allografts for the weight-bearing area of the acetabular rim present limited integration and consecutive resorption, leading to failure of fixation in the long term, modern macro-porous metal augments present a promising alternative [4,5,6,7]. These augments can be combined with different implant components in the means of an augment-and-cage or augment-and-cup construct. A new alternative is a cementless augment-and-modular-cage construct. In these types of implant systems, the augment, in different shapes and sizes, can be combined with a variety of different shells featuring, e.g., cranial straps, different cup orientations, caudal hooks, and more. While this is a new approach to the treatment, first radiographic and clinical results are promising [8]. In cases of severe acetabular bone loss with the need to augment a non-contained defect, a simple press-fit of the augment and component might not be enough. Additional means of fixation can be added in order to improve primary stability. Cranial straps are supposed to provide a surplus of stability by anchoring the acetabular cage or shell to the remaining stable iliac bone stock. Widespread use in clinical practice and extensive clinical research of implant components combined with cranial straps have been conducted and appear favorable [8,9,10,11]. To the best of the authors’ knowledge, no publication describes the surplus of stability achieved by the addition of cranial straps alone in objective and isolated terms. This knowledge, however, is highly important due to the increased damage of soft tissue, especially caused by the mobilization of the gluteal muscle group, required to implant cranial straps. Gluteal insufficiency, risk of damage to the gluteal nerves and vessels, as well as other soft tissue-related complications can be experienced [12]. Another important factor is the amount of relative motion between the individual components of a modular construct. Due to the different prosthesis design (with and without cranial straps), there is a possibility that the individual components of the modular system might react differently to each other and thus may lose stability. To provide an optimal therapeutic solution, modular revision systems need to be thoroughly investigated. The amount of relative motion has been discussed to affect osseointegration of implant components and may cause an increased risk of particle debris, which is associated with implant loosening. A detailed analysis of the possible impact of increased relative motion between components and between bone and the revision system will be provided below.

Therefore, the goal of this study is to evaluate the primary stability of two different designs of a cementless augment-and-modular-cage system with and without the addition of cranial straps in a standardized in vitro setting. As the surrogate parameter for the evaluation of primary stability, the measurement of relative motion between the implant components at all interfaces and the bone (composite hemipelvis model) will be used. The Results will be compared to current literature.

## 2. Materials and Methods

### 2.1. Study Design

We constructed an experimental setup utilizing an acetabular revision component with a trabecular titanium augment (MRS-Titan^®^ Comfort, Peter Brehm GmbH, Weisendorf, Germany) in combination with a large fourth-generation composite left hemipelvis (#3405 Sawbones; Sawbones Europe AB, Malmö, Sweden). Of these constructs (implant and hemipelvis), 12 were prepared in the manner listed below and consecutively divided into two groups (6 with cranial straps (S) and 6 without cranial straps (nS)). Sample size estimation was carried out using G*Power 3.1 Software (Heinrich-Heine-University, Düsseldorf, Germany), [13] using a Mann–Whitney U test for two groups with an expected effect size of *d* = 2.5 and α = 0.05, with power (1-β) = 0.8; we based the chosen values on the well-standardized methods of this experiment and laboratory experience, as well as similar literature [14,15].

### 2.2. Defect Planning and Preparation of Pelvic Models

**Figure 1:**
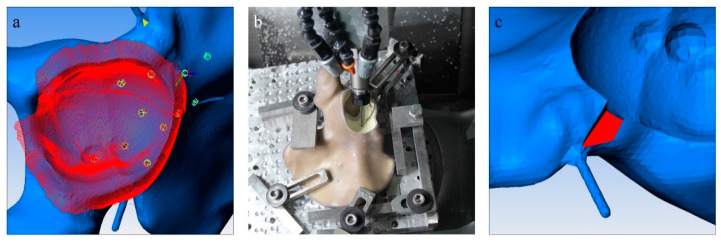
Different steps of the planning and milling process are displayed. (**a**) The segmented bone defect (red) on the digital model of the composite hemipelvis model (blue) and the planned screw holes (green); (**b**) a milling tool in the process of creating the acetabular defect and implant bed as well as the screw holes in the composite hemipelvis model; (**c**) a fabricated gap which enables the addition of a metal rod and a measuring platform to the augment (compare Figure 2).

In order to comply with a frequent clinical setting in cases of acetabular revision arthroplasty, the common morphology of an ADC IIa/Paprosky IIb defect was chosen [3,16]. The segmental bone lesion was located in the superior portion of the acetabular rim with a depth of 1 cm, affecting one-third of the acetabular circumference. To provide a standardized and reproducible defect location, a DICOM format with a layer thickness of 1 mm of the composite hemipelvis was generated through a CT scan using a Philips Brilliance ICT 6000 (Philips, Amsterdam, Netherlands). The DICOM file was subsequently segmented using Materialize Mimics Medical Version 22.0 (Materialise, Leuven, Belgium). The STL-Format was then adapted to include the above-mentioned ADC IIa/Paprosky IIb defect. In addition, the implant bed and screw holes were planned to allow maximum of reproducibility. A gap in the anterior portion of the acetabulum below the inferior anterior iliac spine outside of the weight baring area was added. The purpose of this addition was to accommodate a rod which later would be outfitted with a marker platform (Figure 1c). For the planning process, Pro Engineer Wildfire 2.0 (PTC Inc., Boston, MA, USA) was utilized, and in order to convert the STL-Format into an applicable format, ESPRIT CAD-CAM Build 19.17.170.1385 (Esprit Inc., Camarillo, CA, USA) was employed (Figure 1a). The composite hemipelvis models were then milled in a CNC milling drill (Hermle C12 U Dynamik TNC 640 340590 06 SP7—6 (Berthold Hermle AG, Gosheim auf dem Heuberg, Germany)) with a milling accuracy of 5µm (Figure 1b). Care was taken during planning to include all processing steps into a single working cycle of the CNC milling drill to avoid any deviations due to repositioning of the hemipelvis models. In Figure 1, different steps of the planning and milling process are displayed.

### 2.3. Cementless Augment-and-Modular-Cage Construct and Implantation

The cementless augment-and-modular-cage construct with cranial straps (S) and without cranial straps (nS) was implanted in a standardized fashion by an experienced orthopedic surgeon in accordance with the manufacturer’s instructions and the preceding digital planning. All shells were size 56 mm left combined with a 14 mm A-Augment and either no cranial straps or two cranial straps (45 mm) with 2 screws holes each extending over the superior aspect of the acetabulum to the iliac bone. The A-Augment was pre-operatively modified by the addition of a metal rod which would later carry marker points during the biomechanical analysis. This step was necessary due to the coverage of the augment by the cage. Therefore, in order to enable a valid measurement of relative motion, the metal rod with marker points was used as an extension of the augment. To compare to a clinical setting, a press-fit implantation prior to the insertion of screws was attempted and achieved in all cases. The press-fit of the implant in combination with an uncontained acetabular defect, however it does not offer the same stability as an undisturbed acetabular rim would.

The porous metal augment was attached to the titanium shell and fixated through a singular screw (6 × 12 mm) with 8 Nm torque prior to implantation. After initial press-fit of the cementless augment-and-modular-cage construct, the following screws were added depending on the assigned group (S/nS): S: cranial strap with 4x flathead spongiosa screw 6 × 25 mm, 6 × 30 mm, 6 × 30 mm, 6 × 40 mm; rim with 1 flathead spongiosa screw 6 × 40 mm; dome with 3 flathead spongiosa screw 6 × 25 mm, 6 × 50 mm, 6 × 60 mm; nS: rim with 1 flathead spongiosa screw 6 × 25 mm; dome with 3 screw 6 × 25 mm, 6 × 50 mm, 6 × 60 mm.

Finally, in preparation for the biomechanical analysis, all hemipelvis models with the implanted acetabular component were secured by submerging the posterior ilium into a containment device filled with a liquid two-component casting resin block. In a separate step, the symphysis was also secured into a two-component casting resin block with the addition of a stainless-steel ball on the undersurface. In the biomechanical analysis, this ball is in contact with a metal plate only fixating the symphysis in one plane and therefore allowing multiplanar movement and rotation in order to mimic a more physiological fixation [17,18].

Figure 3 shows the composite pelvis with the implanted modular cage-and-augment construct in the polyurethane setting with the addition of the stainless-steel ball on the undersurface.

### 2.4. Biomechanical Analysis

**Figure 2:**
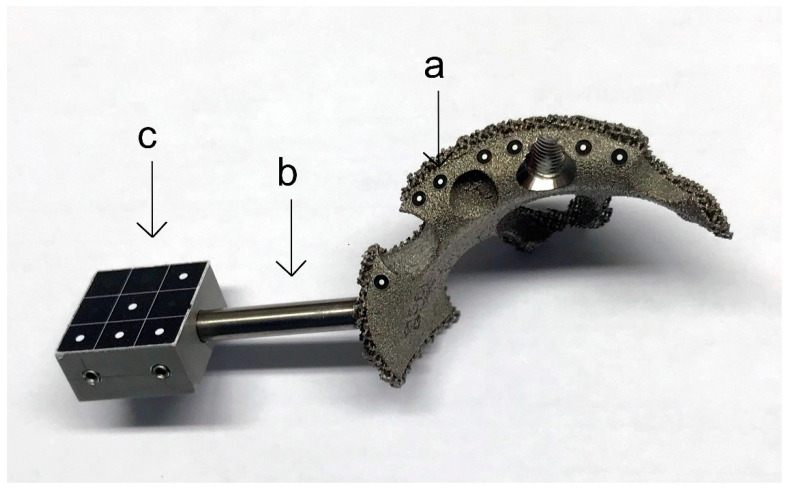
Macro-porous titanium augment with the addition of optical markers (a) and an added metal rod (b) as an extension which holds a metal platform (c) which contains further optical markers. While markers at the implant shell and composite bone were easily attachable (compare Figure 3), the augment was mostly covered by the rim and cranial straps. Therefore, a metal rod was added to the augment as an extension in order to attach a satisfactory number of optical markers.

To enable discrimination and recording of relative movement between the components during loading, the implant-hemipelvis-construct was outfitted with optical markers (uncoded passive white markers with a diameter of 0.8 mm, GOM Item Number: 21874; GOM GmbH, Braunschweig, Germany) along the surface of each component planned to be measured. While markers at the implant shell and composite bone were easily attachable, the augment was mostly covered by the rim and cranial straps. Therefore, the implant manufacturer assembled a specialized augment containing a metal rod as an extension in order to attach a satisfactory number of optical markers (Figure 2).

To record relative movement between the components, an optical measuring system (PONTOS 1; GOM GmbH, Braunschweig, Germany) applying 3D point triangulation detected the optical marker positions in the defined coordinate system. The 3D micromovements in x-, y-, and z-axes were consecutively measured simultaneously between the different interfaces (bone/augment, bone/shell, shell/cup). The dependent variable was the relative movement between the components (measured in μm). The recorded maximal movements of the component augment, shell, bone and cup were calculated using the formula $\left|\vec{R}\right|=\sqrt{x^{2}+y^{2}+z^{2}}$.

**Figure 3:**
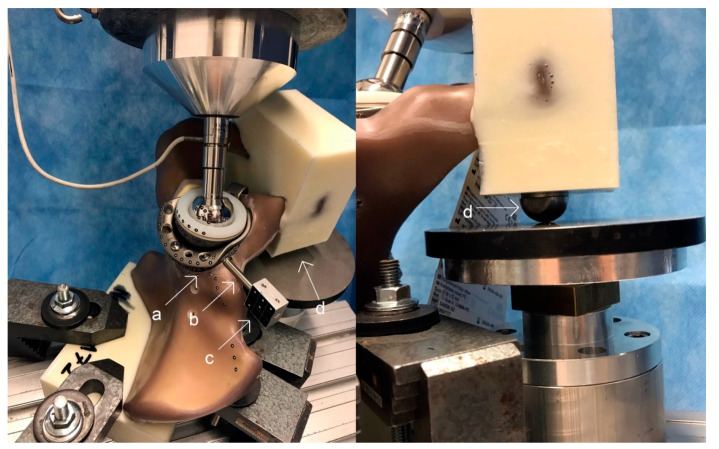
Left: Setup of the implant-hemipelvis-model construct inserted into a material testing machine for the biomechanical stimulation. Visible are multiple optical markers (a) and a metal rod (b) serving as an extension combined with a metal platform (c) in order to attach a satisfying amount of optical markers to the augment. The macro-porous titanium augment is almost entirely covered by the rim portion of the shell. Right: Composite pelvis with the implanted augment-and-modular-cage construct in the polyurethane setting with the addition of the stainless-steel ball on the undersurface (d). Due to this setup the symphysis is only fixed in one degree of freedom and multiplanar movement and rotation is enabled [15].

The biomechanical simulation was carried out by using a material testing machine (MTS Mini Bionix 359; MTS Systems Corporation, Eden Prairie, Minnesota). The implant-hemipelvis-construct set into the material testing machine is displayed in Figure 3.

The vector of the load application coincided with the direction of the greatest load occurring during normal gait [19,20]. Termination criteria were set as fracture of the hemipelvis model.

According to a publication by Bergmann et al., the maximum load during normal walking relates with 233% to the individual’s body weight at 31° of rotation around the x-axis and 5° around the z-axis relative to the described acetabular component system [21].

The experimental body weight was set at 80 kg for each testing (1.8 kN at 100% load). To warrant a proper force closure between the force plate and the implant-hemipelvis-construct, a constant force of 0.2 kN was applied prior to testing. A total of 1000 cycles were applied in a sinusoidal waveform at 1 Hz at each of three load levels: 3% to 30% load (the equivalent of 0.5 kN peak load); then 5% to 50% load (the equivalent of 0.9 kN peak load); and, finally, at 10% to 100% load (1.8 kN peak load). Relative movements between the components were measured at cycles 1 to 50, 51 to 200, 201 to 500, 501 to 800, and 801 to 995 (average and variance).

### 2.5. Statistical Analysis

The statistical evaluation was performed using SPSS Statistics Version 22 (IBM Corp., Armonk, NY, USA). To test for primary stability, the surrogate parameter of relative movement between the components during different loads (measured in µm) was applied. For each interface a separate analysis was carried out (augment/bone, shell/bone, shell/augment, shell/cup). Initial descriptive statistical analysis was applied utilizing the arithmetic mean, range and standard deviation. Normal distribution was assessed through the Kolmogorov–Smirnov and Shapiro–Wilk Test.

A repeated-measures analysis of variance (ANOVA) was calculated for the transformed relative movement (averaged over cycles, as described above, and weighted by the inverse variance of these samples). To validate the ANOVA Mauchly’s sphericity test was used. In cases where sphericity could not be verified the Greenhouse–Geisser correction was applied. As covariates we defined the application of cranial straps (S, nS), force (30%, 50%, and 100% maximal load), and their interaction. The unpaired *t*-test was applied to test for differences between component design (S/nS) according to load when homogeneity of variance could be established. In cases where homogeneity of variance could not be shown, Welch Test was utilized.

Hemipelvis ID was chosen as a random intercept, and post hoc comparisons between S and nS, and S/nS were performed using differences of ‘least-squares’ means [22]. The level of significance for two-tailed *p*-value was set at ≤0.05. 

## 3. Results

**Figure 4:**
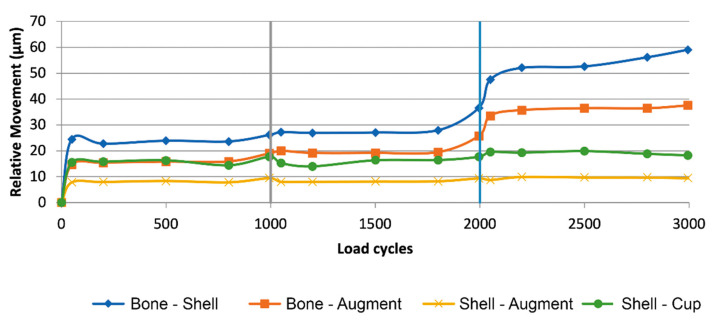
Relative Movement of different interfaces in relation to load (I = 30%, II = 50%, III = 100%) over the accumulation of load cycles in the group without cranial straps (nS), (diamond blue: bone-shell, square orange: bone-augment, cross yellow: shell-augment, circle green: shell-cup). The interface between bone and shell features the largest amount of relative movement with an increase as load levels increase. Followed by the amount of relative motion at the interface of bone and augment which displays an increase with ascending load levels as well. Relative motions at the interfaces of shell and augment and shell and cup remain constant as load levels increase.

**Figure 5:**
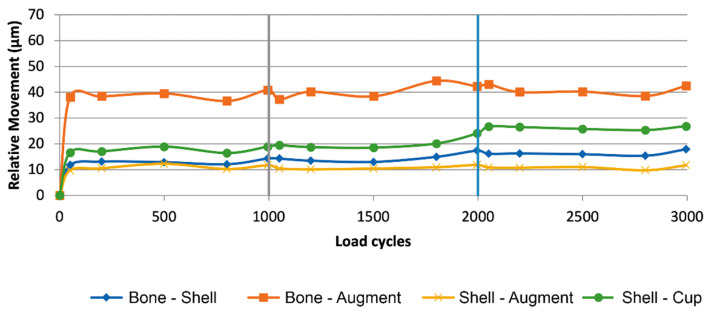
Relative movement of different interfaces in relation to load (I = 30%, II = 50%, III = 100%) over the accumulation of load cycles in the group with cranial straps (S), (diamond blue: bone-shell, square orange: bone-augment, cross yellow: shell-augment, circle green: shell-cup). The interface between bone and augment features the largest amount of relative movement in the construct followed by the interface between shell and cup. Relative motions at the interfaces of shell and augment and shell and bone remain constant as load levels increase.

Table 1 displays the detailed results of the descriptive statistical analysis, the analysis of variance and the unpaired *t*-test/Welch Test. Means of maximal relative movement between the different components with and without cranial straps (nS/s) is displayed in Figure 4 and Figure 5.

Load levels display a significant effect on the amount of relative motion between bone and shell (F(2, 16) = 233,585, *p* < 0.001), bone and augment (F(1.19, 9.55) = 89.52, *p* < 0.001), and shell and cup (F(2, 16) = 65.58, *p* < 0.001). In the interface between shell and augment, no significant effect of load levels on the amount of relative motion could be assessed (F(2, 16) = 3.52, *p* = 0.054).

The group assignment (S/nS) appears to have an effect on relative motion due to significantly differing means in the comparison of bone and shell (F(1, 8) = 219.333, *p* < 0.001), bone and augment (F(1, 8) = 279.101, *p* < 0.001), shell and cup (F(1, 8) = 56.686, *p* < 0.001), and shell and augment (F(1, 8) = 36.357, *p* < 0.001).

**Figure 6:**
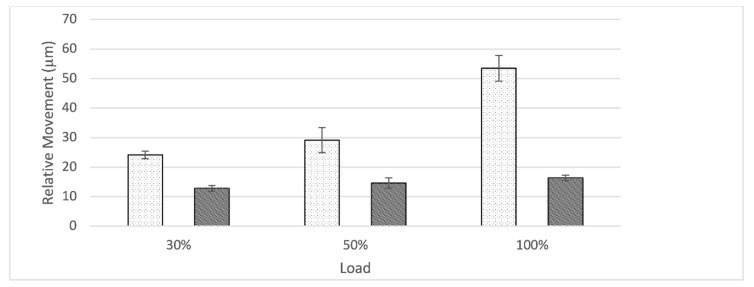
Graph showing relative movement between bone and shell in relation to load divided by group (nS = dotted, S = striped). The S-group displays a constant amount of relative motion as load levels ascend. The amount of relative motion in the nS-group increases as load levels increase.

At the interface between bone and shell, relative movement increased as load level increased for the nS-group while remaining constant throughout for S-group (Figure 6). The nS-group also started with a higher amount of relative movement when compared to the S-group. Relative movement was significantly different between groups for all load levels (30%: t(8) = 15.64, *p* < 0.001; 50%: t(8) = 7.18, *p* < 0.001; 100%: t(4, 36) = 18.61, *p* < 0.001).

**Figure 7:**
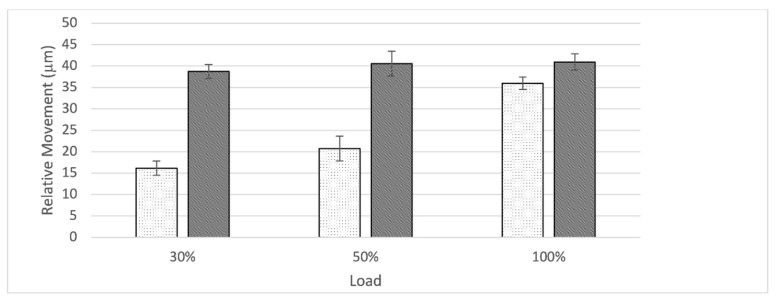
Graph showing relative movement at the interface between bone and augment in relation to load level divided by group (nS = dotted, S = striped). While the S-group started out with a larger amount of relative movement compared to the nS-group, it stayed constant throughout different load levels. The nS-group displayed an increase in relative motion as load levels ascended.

At the interface between bone and augment, relative movement increased as load level increased for the nS-group while remaining constant throughout for S-group (Figure 7). The S- group started with a higher amount of relative movement when compared to the S-group. At 100% load the amount of relative movement between the nS- and S-group aligned. Relative movement was significantly different between groups for all load levels (30%: t(8) = −21.89, *p* < 0.001; 50%: t(8) = −10.87, *p* < 0.001; 100%: t(8) = −4.65, *p* < 0.003).

At the interface between shell and cup, relative movement increased as load level increased for both groups. The S-group started with a higher amount of relative movement when compared to the nS-group. Relative movement was significantly different between groups for the 50% and 100% load level, while the difference at the 30% load level was not significant (30%: t(8) = −2.14, *p* = 0.065; 50%: t(8) = −3.56, *p* < 0.008; 100%: t(8) = −17.32, *p* < 0.001).

**Figure 8:**
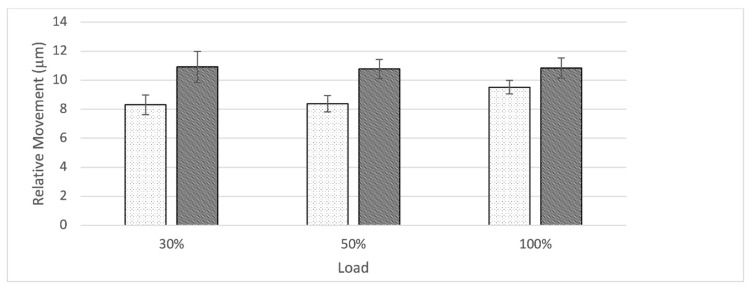
Graph showing relative movement at the interface between shell and augment in relation to load divided by group (nS = dotted, S = striped). Both groups displayed an almost constant amount of relative motion as load level increased. The S-group displays a significantly larger amount of relative motion when compared to the nS-group.

At the interface between shell and augment, relative movement remained constant as load level increased for both groups (Figure 8). The S-group displayed a slightly higher amount of relative movement when compared to the nS-group. Relative movement was significantly different between groups for all load levels (30%: t(8) = −4.56, *p* = 0.003; 50%: t(8) = −6.11, *p* < 0.001; 100%: t(8) = −3.51, *p* < 0.009).

## 4. Discussion

The aim of this publication is to assess the impact of the addition of cranial straps to a cementless augment-and-modular-cage construct on the amount of relative motion between the different components within the system and between implant and bone. Relative movement is an important in vitro measurement in order to assess primary stability and consecutively determine the potential of osseointegration. The conducted in vitro testing brought many significant results to light. In order to properly interpret these findings, the measurement of relative movement needs to be related to a clinical setting. Prior in-vivo animal and human autopsy studies established cut-off values indicating that successful osseointegration occurs at ~40 µm, while relative movement of 150 µm appears to result only in fibrous attachment [23,24]. Although these values cannot be taken as a gold standard, due to various limitations, they underline the need to keep relative movement between implant and bone bed low in order to optimize in-growth conditions.

Besides, proper osseointegration relative movement also affects the implant system as a whole. One of the most relevant reasons for failure of an implant is particle debris with consecutive osteolysis and loosening. Increased relative movement has been associated with increased wear of the implant surfaces resulting in particle debris [25,26]. Due to the modularity of modern revision systems, the number of interfaces increases, and therefore it is reasonable to assume that the chance for particle debris is amplified. Adverse reactions to metal debris (ARMD) and corrosion increase as the number of interfaces increases and have been described for modular revision implants in numerous previous studies [27,28,29,30]. Interestingly, while the amount of relative movement between the shell and the cup increased for both groups, the S-group started with a significantly higher amount of relative movement and remained higher throughout all load levels when compared to the nS-group. This might be due to the increased stiffness of the construct caused by the addition of cranial straps. Therefore, less movement and deformation of the shell is possible and the applied force escapes through an increase of relative motion at the interface between shell and cup. With a maximum amount of relative movement at the highest load level of 26.25 ± 0.66 µm in the S-group, this appears to be a significant effect, however, to access if this actually poses a clinically relevant alteration, further investigation and different methods are required.

While the usefulness of porous metal augments in cases of severe acetabular bone defects has been established, there remains controversy around the best fixation method between augment and shell or cup. In our findings the form-fitting fixation of the augment against the rim-portion of the shell through a single screw proved stable with a constant amount of relative movement across all load levels for both groups. Even though there was a significant difference between relative movements at all load levels to the disadvantage of the S-group, the small difference is not estimated to present a relevant clinical consequence (nS: 9.52 ± 0.46 µm vs S: 10.83 ± 0.70 µm at 100% load) (Figure 7). The group of Beckmann et al. compared three different fixation techniques between a porous metal acetabular component and augments with similar methods. In this study screw fixation, cement fixation and a combination of both were compared in an in vitro setting also utilizing relative motion as the main outcome parameter. Even though a direct comparison due to slight differences in methods might be biased, it is interesting to compare these results. The cementless fixation method of the augment-and-modular cage system appears to be superior presenting a smaller amount of relative motion especially at the 100% load level (nS: 9.52 ± 0.46 µm vs S: 10.83 ± 0.70 µm at 100% load; Beckmann et al.: cement: 11.3 ± 1.9 µm, screw plus cement: 12.6 ± 4.0 µm, screw 31.4 ± 16.6 µm at 100% load) [15]. Further studies are needed to investigate these findings in a controlled manner.

The initial design of straps or flanges surfaced in reconstruction rings/cages which would bridge an acetabular bone defect in order for the added bone graft below to be protected until it would eventually consolidate. Due to vast improvements in implant design, modern revision systems offer a variety of different modes of fixation such as cranial or dorsal straps/flanges, iliac pegs, caudal hooks, and iliac lag screws. While there are plenty publications assessing the clinical use of implant systems (e.g., cranial socket system, augment-and-cage, augment-and-modular-cage, and individualized partial pelvic implants) which utilize cranial straps, to the knowledge of the authors, no studies exist which objectively quantify the potential gain in stability achieved in a clinical or an in vitro setting. The main objective of this publication is to evaluate if cranial straps provide additional primary stability. To answer this question, the contact surfaces of the implant to the bone are of major concern. In the interface between shell and bone, the addition of cranial straps provided a lower amount of relative movement which remained constant across all load levels. In comparison, in the absence of cranial straps, relative movement started out higher and even increased as load levels increased. Therefore, in addition to the initial design intend, cranial straps seem to provide additional stability to the shell. Especially at the 100% load level, relative movement between the groups were significantly different (nS: 53.48 ± 4.36 µm; S: 16.36 ± 0.93 µm) (Figure 6). In the light of these results, it is reasonable to conclude that cranial straps add additional stability to the implant shell. However, to evaluate the clinical impact of this added stability, further clinical studies are needed.

Finally, the analysis of the interface between augment and bone provided another interesting insight. Even though relative movement between components within the S-group remained constant over all load levels, it started out significantly higher when compared to the nS-group. The nS-group, starting out significantly lower, displayed an increase across ascending load levels and finally almost aligned with the S-group at the 100% load level (Figure 8). Due to the significant but slight difference between groups at 100% load and the constant course of relative movement in the S-group, we interpret these findings as not being evidence of a decrease of stability due to the addition of cranial straps (100% Load; nS: 35.96 ± 1.46 µm; S: 40.92 ± 1.89 µm). Furthermore, these values are within the established threshold for successful osseointegration [23,24].

Our methods present several limitations. We chose to utilize composite pelvic models instead of cadaveric bone in this in vitro setup in order to decrease inter-specimen variability and improve availability and ease of use. In addition, the application of a consistent acetabular defect in cadaveric bone with varying anatomical setup and bone density is considered challenging. Even though our choice enabled a reproducible in vitro experiment, correlation with the clinical situation might be limited due to different biomechanical properties such as strain distribution and overall stiffness in a fourth-generation composite model [31]. 

The impact of the addition of cranial straps was only assessed for a specific augment-and-modular-cage revision system. Therefore, a generally valid statement for the many available systems utilizing cranial straps cannot be obtained as of now. We strongly encourage the application of the presented methods to other implant systems and even different set ups of the same augment-and-modular-cage revision systems by other groups. The applied defect morphology (ADC IIa/Paprosky IIb) was chosen due to a wide incidence in the daily reality of hip revision arthroplasty [3,16]. Different defect morphologies and consecutively different choice of augmentation may lead to alternative results and need further investigation as well. Furthermore, the chosen mode of load application, as described in methods, did not replicate the entire cyclical pattern or normal walking with loading on different areas of the acetabulum in differing degrees.

We hope our study inspires future research into the subject. Especially in vivo studies of different fixation techniques between the components within a modular system and the impact of different configuration of that system should be evaluated.

## 5. Conclusions

Our study evaluated the gain in primary stability through the addition of cranial straps to an augment-and-modular-cage revision system. We utilized a reproducible in vitro setup with composite hemi-pelvis models and measured relative movement between individual components in addition to relative movement between components and the bone as a surrogate parameter for primary stability.

We conclude a significant increase of primary stability between the shell and the bone through the addition of cranial straps. Relative motion between components (shell/cup) increases through the addition of cranial straps. A clinical impact of this finding is uncertain and requires further investigation. Finally, the cementless fixation of the augment against the rim-portion of the shell appears stable and compares favorably to prior investigation of different fixation techniques.

## Figures and Tables

**Table 1:** Descriptive statistic evaluation of relative movement between components, ANOVA, and unpaired *t*-test. Means of maximal relative movement (µm) are provided with standard deviation relative to group (nS/S); Load levels (%) relative to interface.

Load, %	Mean Relative Movement (RM), µm ± SD		ANOVA RM—Load		ANOVA Group—RM		Unpaired T-Test/ Welch Test	
	AmC without Cranial Straps (nS)	AmC with Cranial Straps (S)	F	Sig.	F	Sig.	T	Sig.
Shell—Augment			3.52	*p* = 0.054	36.357	*p* < 0.001		
30	8.32 ± 0.68	10.92 ± 1.08					−4.559	*p* < 0.003
50	8.38 ± 0.56	10.77 ± 0.67					−6.110	*p* < 0.001
100	9.52 ± 0.46	10.83 ± 0.70					−3.507	*p* < 0.009
Bone—Augment			89.520	*p* < 0.001	279.101	*p* < 0.001		
30	16.17 ± 1.67	38.72 ± 1.60					−21.798	*p* < 0.001
50	20.72 ± 2.85	40.55 ± 2.92					−10.867	*p* < 0.001
100	35.96 ± 1.46	40.92 ± 1.89					−4.652	*p* < 0.003
Bone—Shell			233.585	*p* < 0.001	219.333	*p* < 0.001		
30	24.186 ± 1.28	12.84 ± 1.00					15.637	*p* < 0.001
50	29.15 ± 4.19	14.61 ± 1.71					7.183	*p* < 0.001
100	53.48 ± 4.36	16.36 ± 0.93					18.607	*p* < 0.001
Shell—Cup			65.582	*p* < 0.001	56.686	*p* < 0.001		
30	15.94 ± 1.20	17.59 ± 1.23					−2.138	*p* < 0.065
50	15.93 ± 1.39	20.17 ± 2.27					−3.557	*p* < 0.008
100	19.15 ± 0.64	26.25 ± 0.66					−17.316	*p* < 0.001

## Data Availability

Data are available on request due to privacy restrictions.
